# Screening of CCDC43 molecular partners by BioID2-based proximity labeling

**DOI:** 10.55730/1300-0152.2744

**Published:** 2025-03-17

**Authors:** Merve TUZLAKOĞLU ÖZTÜRK

**Affiliations:** Department of Molecular Biology and Genetics, Faculty of Science, Gebze Technical University, Gebze, Kocaeli, Turkiye

**Keywords:** CCDC43, BioID, mass spectrometry

## Abstract

**Background/aim:**

CCDC43 (coiled-coil domain containing protein 43) is a eukaryotic protein that contains alpha-helical domains in its structure, consistent with the family to which it belongs. It is predominantly located in the cytosol of the cell. CCDC43 protein has been shown to play a role in cell proliferation, invasion, metastasis, and epithelial–mesenchymal transition in gastric and colorectal cancer types. The fundamental function and cellular interaction network of this protein, which is known to have varying expression levels in various cancer types, has not yet been fully elucidated. Here, we conducted a proximity-dependent biotin identification (BioID2) screening approach for CCDC43 to uncover proximity interactors.

**Materials and methods:**

Using mass spectrometry of streptavidin pull-down of biotinylated proteins with filtering approaches, we identified candidate protein interactors for CCDC43.

**Results:**

We suggest an association between CCDC43 and RNA-binding proteins based on all our biological replicates. The strongest candidate for the interactome is YBX1, a highly conserved cold shock domain and nucleic acid-binding protein that has multiple essential functions in the cell.

**Conclusion:**

These findings suggest that CCDC43 may play a role in critical pathways within the cell.

## 1. Introduction

The coiled-coil domain containing 43 protein (CCDC43), belongs to the family of coiled-coil domain-containing proteins, which are characterized by the presence of domains containing structural motifs consisting of two or more coiled alpha-helices (Ford and Fioriti, 2020). Despite their structural similarities, proteins belonging to this family play important key roles in a wide variety of processes in the cell (Priyanka and Yenugu, 2021). Human CCDC43 consists of 224 amino acids and is encoded by the corresponding gene located on chromosome 17q21.31 (Wang et al., 2018). Although CCDC43 is expressed in various tissues and cell types, previous studies have focused on its alteration in different types of cancer rather than its fundamental functions in the cell. As demonstrated in the extant literature, research focusing on CCDC43 and its correlation with various cancer types has revealed a significant upregulation of this protein’s mRNA levels in numerous cancerous tissues, including those of the lung, head and neck, cervix, adrenal gland, stomach, liver, esophagus, bladder, prostate, ovary, breast, vagina, testis, and colon (Wang et al., 2018).

CCDC43 is known to play an important role in studies of gastric cancer (GC). Increased expression of CCDC43 can lead to proliferation, invasion, and metastasis in GC cells (Tao et al., 2023). In gastric cancer, CCDC43 has been shown to colocalize with four and a half LIM domain protein 1 (FHL1), but these two proteins act as antagonists. While CCDC43 functions as an oncogene in gastric cancer, FHL1 promotes apoptosis of GC cells (Tao et al., 2024). It has also been reported that increased expression of CCDC43 in advanced oral squamous cell carcinoma (OSCC) is associated with reduced antitumor immunity and consequently contributes to the metastasis of OSCC cells (Wang et al., 2022). In studies of the molecular functions of CCDC43, it is crucial to determine which proteins it interacts with to see the whole picture.

Many cellular proteins interact with each other to fulfill their biological functions and to extend this functional repertoire. Protein–protein interactions (PPIs), together with the cellular biological regulatory system for proteins, are essential elements in regulating the formation of functional complexes in the cell. Therefore, understanding how proteins interact with other proteins is critical. PPIs are context-dependent; they can be diverse and transient, and they show remodeling under different conditions. They are mainly involved in cellular pathways and their control. The identification of protein–protein interactions is performed using several technologies, including low- and high-throughput techniques. These methods include the yeast-two-hybrid (Y2H) system, affinity purification combined with mass spectrometry (AP-MS), luminescence-based mammalian interactome mapping (LUMIER), mass-spectrometry (MS)-based methods (affinity purification (AP), cofractionation (CF), proximity labeling, and cross-linking), atomic force microscopy, surface plasmon resonance, Förster resonance energy transfer (FRET), bioluminescence resonance energy transfer (BRET), bimolecular fluorescence complementation (BIFC), and superresolution microscopy (Yugandhar et al., 2019; Benz et al., 2020; Richards et al., 2021). Furthermore, using methods combined with AI-based approaches such as AlphaFold, protein–protein interactions can be detailed at the molecular level (Greenblatt et al., 2024). Since intracellular proteins are regulated by highly complex systems involving multiple cascades, the use of advanced or even multiple technologies can help us understand cellular protein regulation.

The development of proximity labeling technology has facilitated the identification of compartmental proteomes and protein interactions (Bosch et al., 2021). BioID (proximity-dependent biotin identification) is a special version of proximity labeling technologies based on coimmunoprecipitation of biotinylated proteins by streptavidin interaction (Roux et al., 2012). This method can be used for strong as well as weak and transient interactions of the target protein. It also allows us to take ‘snapshots’ of protein–protein interactions. The first version of BirA in BioID originated from *Escherichia coli* biotin ligase. The reduced-size mutant BirA, termed BioID2, is the second generation of BirA. The reduced size of the enzyme increased its activity, and it is derived from *Aquifex aeolicus* biotin ligase ( Kim et al., 2016 ). Compared to BioID, BioID2 has a lower biotin requirement but more accurate targeting and localization when the protein of interest is a fusion protein (May et al., 2020).

In this study, we screened for potential CCDC43-interacting proteins using the BioID assay. We found YBX1 to be a strong candidate interactor CCDC43. Furthermore, RNA-binding and splicing factors also appear to be candidates for CCDC43 interaction.

## 2. Materials and methods

### 2.1. Cell lines and culture

Human embryonic kidney 293T (HEK293-T) cells were cultured in Dulbecco’s modified Eagle’s medium (DMEM) (Gibco, Waltham, MA, USA) supplemented with 10% fetal bovine serum (FBS) and 1% penicillin/streptomycin. The cells were maintained at 37 °C in a humidified incubator containing 5% CO_2_. Cells were transiently transfected with the BioID2 plasmid constructs using polyethylenimine (PEI) transfection reagent.

To create the BioID2 fusion construct, the full-length CCDC43 gene was PCR-amplified using the primers forward (5′ → 3′): GAA TTC ATG GCG GCG CCC AGC GAA GTG and reverse (5′ → 3′): AAG CTT TTA TCG CTT TCG CTC CCC TCT, and cloned into pcDNA3.1 Myc-BioID2 plasmid (Addgene #74223, Watertown, MA, USA). The created construct was validated by Sanger sequencing.

### 2.2. Western blotting

To validate the expression of the BirA*–CCDC43 fusion protein, cells were seeded in a 6-well plate for 24 h (mock, BirA*–empty vector control, and BirA*–CCDC43). Cells were treated with 50 μM biotin at the final concentration and incubated overnight for 16–18 h. Cells were then lysed with lysis buffer containing protease inhibitors (50 mM Tris, pH 8.0; 150 mM NaCl; 1% NP-40), and samples were electrophoretically separated by SDS-PAGE (4%–12%) and transferred to a nitrocellulose membrane. Nitrocellulose membranes were blocked with buffer containing 5% nonfat dry milk or bovine serum albumin (BSA) in TBS-T. The membranes were then incubated with anti-Myc antibody overnight at 4 °C (1:1000), and with HRP-conjugated streptavidin (1:10,000) for 1 h at room temperature. Membranes were then washed three times with 1X PBS for 5 min. For anti-Myc antibody, a secondary antibody was used after the washes, and imaging was performed. Membranes were visualized using the ChemiDoc XRS system (Bio-Rad Laboratories, Hercules, CA, USA) after treatment with ECL chemiluminescent substrate.

### 2.3. BioID

For the BioID pull-down assay, 5 million HEK293-T cells were grown in four 15 cm plates for 24 h. The cells were transfected with 15 μg of BirA*–empty and BirA*–CCDC43 vectors. In parallel, the cells were mock-transfected with PEI to establish a control group. After 24 h of transfection, cells were treated with biotin (50 μM) for 17 h prior to processing.

Cells were washed twice with 1X PBS and lysed in 0.540 mL of lysis buffer (50 mM Tris, pH 7.4; 500 mM NaCl; 0.4% sodium dodecyl sulfate (SDS); 1 mM dithiothreitol (DTT); and 1X protease inhibitor cocktail). Subsequently, 0.2 mL of 20% Triton X-100 was added. Lysates were sonicated for 2 min using a Branson sonicator (Branson Ultrasonics, Danbury, CT, USA) to disrupt visible aggregates (30% amplitude, 1 min ON; 2 min OFF). Next, 2.52 mL of cold lysis buffer was added to the sonicated lysate. The sample was centrifuged at 15000 × *g* for 15 min, and the supernatant was added to preequilibrated streptavidin beads (New England Biolabs, Ipswich, MA, USA). The mixture was rotated overnight at 4 °C. The next day, beads were collected using a DynaMag rack (Thermo Fisher Scientific, Waltham, MA, USA) and washed twice with 2% SDS for 10 min, followed by a wash with 0.1% sodium deoxycholate, 1% Triton X-100, 500 mM NaCl, 1 mM ethylenediaminetetraacetic acid (EDTA), and 50 mM HEPES (pH 7.5) for 10 min. Next, beads were washed with 250 mM lithium chloride (LiCl), 0.5% NP-40, 0.5% sodium deoxycholate, 1 mM EDTA, and 10 mM Tris (pH 8.0) for 10 min, followed by a wash with 50 mM Tris (pH 7.4). Ten microliters of the suspension were used for Western blotting to check biotinylation. The beads were finally resuspended in 100 μL of 50 mM NH_4_HCO_3_, prior to mass spectrometry analysis.

### 2.4. Mass spectrometry

Proteins were digested on streptavidin beads. Data were acquired on a Q Exactive HF Quadrupole-Orbitrap mass spectrometer (Thermo Fisher Scientific, Bremen, Germany) coupled with nanoliquid chromatography (nLC-MS/MS) using the UltiMate 3000 NCS-3500RS system (Thermo Dionex, Sunnyvale, CA, USA) at a flow rate of 300 nL/min. Proteins were identified with a 0.01% false discovery rate (FDR). Two biological replicates and three technical acquisitions were performed.

### 2.5. Bioinformatic analyses and statistics

At least two technical replicates from each biological replicate were identified, containing at least one unique peptide for CCDC43 samples. A value of zero indicated that no hits were identified for that study. If any CCDC43 sample had a value of zero for a given hit, that value and its corresponding value in empty vector (EV) were excluded from the calculations. This ensured the same sample size for CCDC43 and EV values, and correct fold changes and p-values were calculated. The high-confidence interactors list from proteomic data was analyzed using the Enrichr web tool (Chen et al., 2013).

## 3. Results and discussion

**Figure 1:**
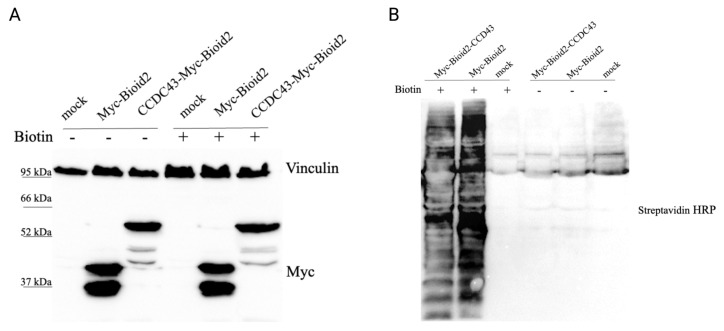
Validation of Myc-BirA and Myc-BirA–CCDC43 expression. A) Immunoblot of nonbiotinylated and biotinylated proteins from cells expressing Myc-BirA and Myc-BirA-CCDC43, alongside untransfected control cells. B) Biotinylation of proteins detected with HRP-conjugated streptavidin.

In the present study, a BioID-based approach was employed to identify proximal interactors of CCDC43 through mass spectrometric analysis. A smaller mutant form of the biotin protein ligase enzyme from *Aquifex aeolicus* was utilized as a tool to biotinylate and identify neighboring proteins interacting with CCDC43. For this purpose, CCDC43 was cloned into the BioID2 vector, which was tagged with a Myc label and mutant biotin ligase at the N terminus. To control the expression levels of the resulting fusion proteins, HEK293-T cells were transiently cotransfected with Myc-tagged BioID2 (empty vector) and BioID2-CCDC43-expressing vector. Biotin was added to induce biotinylation in cells, and the expression levels of biotinylated and nonbiotinylated cells were analyzed by Western blot. Western blotting showed Myc-tagged bands at the appropriate size for the BioID2-CCDC43 fusion protein (53 kDa) and the control BioID2 empty vector (27 kDa) (Figure 1A).

BioID is an enzyme that produces biotinyl-AMP (bioAMP) by utilizing ATP and biotin as substrates. In contrast to the wild-type (WT) enzyme, BioID exhibits a unique property of releasing bioAMP rather than retaining it at its active site. This results in the nonenzymatic biotinylation of lysine residues on proteins within a radius of approximately 10 nm (Schopp et al., 2017; Oostdyk et al., 2019). The identification of proteins that undergo this modification can be facilitated by mass spectrometry using a streptavidin-based pull-down method.

The induction of this process was achieved by the addition of 50 μM biotin to the culture, which was maintained for 17 h. Thus, the streptavidin-HRP blotting membrane revealed the presence of biotinylated proteins in a wide range of molecular weights in each lysate, with an increased level of biotinylation of the respective bait protein (Figure 1B).

**Figure 2:**
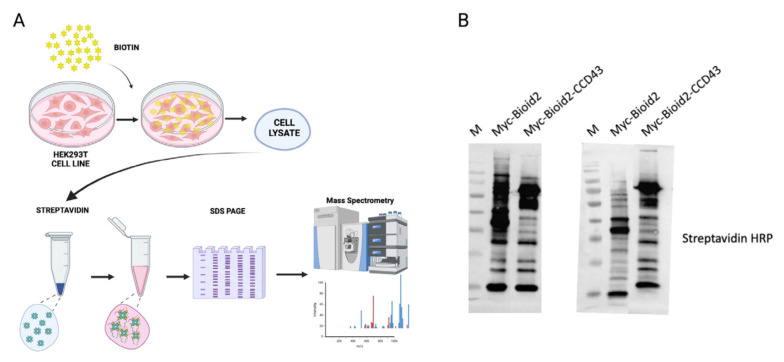
Streptavidin pull-down assay. A) Workflow of BioID streptavidin pull-down. B) Control Western blotting of affinity-purified proteins. M: Protein marker.

We subsequently conducted a streptavidin pull-down experiment (Figure 2A). Myc-BioID2 was used as a negative control for biotinylated proteins. Cells were harvested, and proteins were collected by streptavidin pull-down and subsequently subjected to mass spectrometry for identification of proximity interactors of CCDC43.

**Figure 3:**
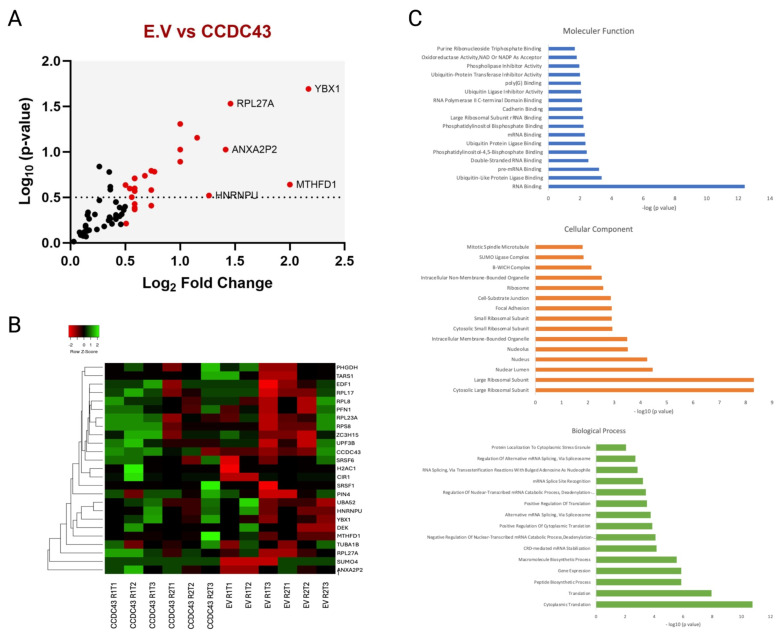
Analysis of mass spectrometry data. a) Log_2_ ratios of the major list plotted against –log10 p-values. b) Relative enrichment of CCDC43 interactors compared to the empty vector. c) Gene ontology analysis.

To check the biotinylation status of each replicate, streptavidin antibody was used as previously mentioned (Figure 2B). The abundance of proteins was then assessed using spectral counting, and candidate proteins were selected by comparing their enrichment in the CCDC43 fusion protein versus those overexpressing only BirA. Subsequently, we collected all runs (a total of two biological replicates and six technical replicates) for statistical analysis. In our approach, a methodology was developed for the filtration of nonspecific proteins appearing in both the empty vector and CCDC43-BioID2 replicates. This filtration was conducted according to log_2_ fold change to a threshold of 0.5, and proteins below this value were removed from the candidate protein lists. The resulting list was plotted to compare proteins enriched in CCDC43 with those found in the control BioID2 samples (Figure 3A). The results indicated that YBX1, RPL27A, ANXA2P2, MTHFD1, and HNRNPU proteins were the strongest interactors of CCDC43. Subsequently, heat maps were applied to the obtained data. The heat map, which was generated by ranking the hits according to log_2_ fold change, provided quantitative data showing the peptide abundance in each read (Figure 3B).

In order to ascertain the molecular context and cellular functions of the high-scoring interacting proteins, the main proximity interaction list was subjected to Gene Ontology (GO) enrichment analysis (Figure 3C). The results of this analysis indicated that CCDC43 was mostly associated with ribosomal proteins. Although studies on CCDC43 have not yet provided definitive information on its intracellular functions, it is suggested in databases as a potential RNA-binding protein. It is noteworthy that a significant proportion of these proteins are nucleic acid-binding proteins. However, they are also capable of multivalent interactions under different conditions with the principle of liquid-liquid phase separation in the cell. CCDC43, like other coiled-coil proteins, carries structural signatures in accordance with this principle. However, it is possible that this tendency is condition-dependent.

Despite the absence of FHL1 protein (four and a half LIM domain 1) in BioID2-CCDC43, a finding that contrasts with previous reports of this protein as a CCDC43 interactor, the potential specificity of this interaction to gastric cancer remains a subject of interest. This is further underscored by the observation of Chen et al. (2022) of a negative correlation between CCDC43 and FHL1 expression levels in GC cells.

Our approach identified YBX1 as the most enriched biotinylated protein, while CCDC43 is not among previously identified YBX1 interactors. Surprisingly, among the interaction partners of YBX1 in the STRING database, HNRNPU—one of the most significant interaction targets identified for CCDC43—is found (Szklarczyk et al., 2023). YBX1, a multifunctional protein belonging to the highly conserved Cold Shock Domain protein family, plays a pivotal role in various cellular functions, including the regulation of transcription, mRNA stability and splicing, DNA repair, and translation regulation (Zhang et al., 2024). Recent studies have identified a link between YBX1 and the regulation of malignant phenotypes in various tumor types (Dinh et al., 2024). While YBX1, as a transcription factor, is capable of binding to DNA, it also interacts with a significant number of mRNAs. The phosphorylation status of YBX1 serves as a crucial regulator of these interactions. The majority of YBX1 is found in the cytoplasm, where it is localized; however, following phosphorylation, it migrates to the nucleus to modulate the transcription of various genes and to participate in DNA repair. In the literature, numerous studies report that upregulation and nuclear localization of YBX1 are considered poor prognostic indicators in various cancer types (Yin et al., 2022).

Heterogeneous nuclear ribonucleoproteins (hnRNPs) are a family of nuclear RNA-binding proteins involved in stages of RNA metabolism (Geuens et al., 2016). The majority of hnRNP studies in the literature have focused on their role in the processing of newly transcribed or nascent RNA to form mature mRNA molecules. However, it is hypothesized that hnRNPs may also have roles in chromatin and thus nuclear structure. While hnRNPs are known to be RNA-binding proteins that interact with RNA species in the nucleus, it has been demonstrated that most of the proteins in this family act through phase separation and can form condensates that may be detrimental to normal cell function (Lee et al., 2022).

This family of proteins is significant to cells, and HNRNPU (SAF-A) is a notable member. It plays a crucial role in the organization of chromatin by interacting with RNA to form a scaffold-like structure (Marenda et al., 2022). It is noteworthy that HNRNPU, like YBX1, is a protein whose gene expression is altered in numerous types of cancer. Like other family members, it contains an RNA-binding domain (RGG repeat), while also displaying a role in DNA repair.

In our major list of potential targets above the threshold value in the CCDC43 interactome, members of the serine/arginine-rich (SR) protein family—another important protein family with high evolutionary conservation—appear to be of particular interest. SRSF1 and SRSF6 are proteins that play an important role in RNA alternative splicing and are known to shuttle between the nucleus and cytoplasm. Like other proteins of the spliceosome machinery, the variability in expression levels of these RNA-binding proteins is particularly relevant in colorectal carcinomas for SRSF6 and in prostate and lung carcinomas for SRSF1 (Li et al., 2023).

To comprehend the function of specific genes in cancer and their role in disease progression and proliferation, several studies have investigated their cellular expression levels. Nevertheless, it is also imperative to understand the mechanisms of protein-protein interactions, in addition to the role of individual proteins in disease progression, to elucidate commonly affected genes and pathways. Consequently, this facilitates the identification of new cancer-specific targets and pathways. In this study, we investigated the CCDC43 protein network by examining proximal interactors using the BioID2 assay.

Thus, we propose that CCDC43 is a critical cellular protein associated with RNA-binding proteins and related ribosomal activities, and may play a regulatory role in the cell in the context of protein translation and the splicing machinery. It is critical to understand the molecular interactions and functional significance of CCDC43 to elucidate its role in cellular physiology and pathology. This protein may emerge as a promising target for therapeutic intervention.
